# High-Dimensional Immunophenotyping of Plasma-Derived Small Extracellular Vesicles in Pancreatic Cancer: An Exploratory Proof-of-Principle Study

**DOI:** 10.3390/biom16070942

**Published:** 2026-06-24

**Authors:** Sabrina Sulzer, Johanna Lisa Becker, Laura Domogalla, Volker Ellenrieder, Matthias Schulz, Markus Maulhardt, Alexander Casimir Angleitner, Judith Büntzel

**Affiliations:** 1 Department of Gastroenterology, Gastrointestinal Oncology and Endocrinology, University Medical Center Göttingen, 37075 Goettingen, Germany; sabrina.sulzer@med.uni-goettingen.de (S.S.);; 2 Department of Hematology and Medical Oncology, University Medical Center Göttingen, 37075 Goettingen, Germanyalexander.angleitner@med.uni-goettingen.de (A.C.A.)

**Keywords:** pancreatic ductal adenocarcinoma, small extracellular vesicles, liquid biopsy, immunophenotyping, plasma biomarkers, machine learning

## Abstract

Pancreatic ductal adenocarcinoma (PDAC) is increasingly recognized as a systemic malignancy, characterized by profound alterations in tumor–host interactions. Small extracellular vesicles (sEVs) in peripheral blood may reflect these alterations and represent a promising minimally invasive source of biomarker information. In this proof-of-principle study, plasma-derived sEVs from patients with PDAC, healthy controls, and a comparative cohort with neuroendocrine lung cancer (NLC) were isolated by differential ultracentrifugation and characterized by western blotting and nanoparticle tracking analysis. Surface marker profiling was performed using the MACSPlex EV Kit IO, followed by univariate, multivariate, and machine-learning-based analyses. PDAC samples exhibited a distinct sEV immunophenotype with coordinated enrichment of angiogenesis-related markers (including CD105 and CD146), immune-regulatory markers (including CD25 and CD40), the coagulation-related marker CD142 and the invasion-associated marker MCSP. Principal component analysis, hierarchical clustering, and Random Forest classification showed exploratory separation of PDAC patients from healthy controls and NLC, supporting the presence of disease-specific vesicle surface marker patterns. In a very small subset of paired samples, descriptive longitudinal analyses illustrated measurable intra-individual changes during chemotherapy. Plasma sEV immunophenotyping is a technically feasible approach for capturing systemic disease-associated alterations in PDAC and provides a foundation for future biomarker-oriented validation studies.

## 1. Introduction

Pancreatic cancer is one of the most aggressive malignancies and is associated with a particularly poor prognosis. Following lung and colorectal cancer, it represents the third leading cause of cancer-related mortality worldwide [1]. Due to its increasing incidence, pancreatic cancer is projected to become the second leading cause of cancer-related death in Germany by 2030 [2]. The most common histological subtype is pancreatic ductal adenocarcinoma (PDAC), which arises from the exocrine pancreas [3].

Currently, complete surgical resection followed by adjuvant chemotherapy represents the only potentially curative treatment option. However, this is feasible in less than 20% of patients at the time of diagnosis [4]. Even after successful resection, the 5-year overall survival rate remains low at approximately 20% [5,6]. For patients with good performance status (Eastern Cooperative Oncology Group (ECOG) status 0–1), systemic therapy with the FOLFIRINOX regimen (folinic acid, fluorouracil, irinotecan, and oxaliplatin) is recommended in both the (neo)adjuvant and palliative settings [7].

These unfavorable outcomes are largely driven by the absence of effective strategies for early detection and disease monitoring. As a result, PDAC is frequently diagnosed at an advanced stage, underscoring a fundamental limitation in current diagnostic approaches. In addition, commonly used biomarkers such as Carbohydrate-Antigen 19-9 (CA19-9) have important limitations. This is particularly relevant in patients who are CA19-9-negative at baseline (for example, due to a Lewis antigen-negative phenotype), which restricts the utility of CA19-9 for disease monitoring [8].

These limitations underscore the urgent need for novel biomarkers that are non-invasive, biologically informative, and applicable in a longitudinal setting. In recent years, liquid biopsy approaches have emerged as promising tools to capture tumor-derived information from peripheral blood. Among these, extracellular vesicles (EVs), including small EVs (sEVs, also called exosomes), have gained increasing attention: They play a central role in intercellular communication and reflect the molecular and functional state of their cell of origin [9]. EVs are actively secreted by tumor cells and carry a complex cargo of proteins, lipids, and nucleic acids, thereby mirroring both tumor-intrinsic characteristics and features of the tumor microenvironment [10]. With a diameter of 50–150 nm, sEVs are particles of endosomal origin secreted into various bodily fluids such as saliva, urine or blood [9].

In PDAC, sEVs have been implicated in key oncogenic processes, including epithelial–mesenchymal transition, angiogenesis, immune modulation, and pre-metastatic niche formation, highlighting their role in tumor progression and systemic disease dissemination [11,12]. Previous studies have demonstrated that sEV-derived biomarkers can distinguish PDAC from benign pancreatic diseases with high accuracy and, in some cases, outperform conventional markers such as CA19-9. However, many of these approaches rely on invasive sampling methods, such as pancreatic juice or cyst fluid. This limits their applicability for repeated measurements and longitudinal disease monitoring. In contrast, blood-derived EVs offer a non-invasive and repeatable alternative that may better capture systemic tumor dynamics over time [13,14,15,16,17].

Taken together, these observations suggest that EV may provide a comprehensive, system-level representation of tumor biology that extends beyond single-marker approaches. In line with the evolving hallmarks of cancer framework, which increasingly conceptualizes cancer as a multidimensional, organism-wide disease process rather than a purely localized lesion [18]. Such an integrated readout from sEVs therefore appears particularly well suited to capture the systemic nature of malignant transformation and progression. In the present study, we focused specifically on sEVs rather than on the entire spectrum of circulating EVs. This decision was primarily driven by methodological and translational considerations. We aimed to use a standardized, commercially available, bead-based multiplex platform that is designed for exosome/sEV-enriched preparations and allows high-dimensional surface marker profiling in a reproducible and high-throughput-compatible manner. Therefore, our workflow is deliberately enriched for sEVs while reducing larger vesicles and cellular debris. This focus should not be interpreted as implying that larger EV subpopulations are biologically irrelevant; rather, it reflects the technical scope of the present proof-of-principle study.

We therefore investigate circulating sEVs as a liquid biopsy-based tool in PDAC. Specifically, we aim to characterize sEV immunophenotypes and explore their potential as composite biomarkers for disease detection, differentiation from non-malignant conditions, and longitudinal monitoring under systemic therapy. By focusing on multidimensional marker patterns rather than individual molecules, we seek to provide a more integrative understanding of tumor-associated processes and their clinical relevance.

## 2. Materials and Methods

*Patient recruitment.* The study was conducted in accordance with the Declaration of Helsinki and approved by the local ethics committee (project no. 3/2/14, approval date 17 March 2014). Case allocation followed an approximately 2:1 ratio of PDAC patients to healthy controls (*N* = 22 versus *N* = 12). Additionally, 18 patients with neuroendocrine lung cancer (NLC, 17 small cell lung cancer, 1 large cell lung cancer) were added to the sample pool for subsequent comparison of PDAC and NLC samples. Samples were collected between October 2014 and February 2026. Eligible cases had histologically or cytologically confirmed PDAC or NLC. To minimize contamination with apoptotic bodies, only patients without current chemotherapy were included, defined as either chemotherapy-naïve or at least 7 days after the last cycle. In the NLC cohort, this criterion was fulfilled by 17 of 18 patients. One patient was sampled one day after chemotherapy and was retained, given the exploratory nature of the tumor-entity comparison. Patients were recruited at the Department of Hematology and Medical Oncology and the Department of Gastroenterology, Gastrointestinal Oncology and Endocrinology of the University Medical Center Göttingen. Control samples were obtained from hospital staff, healthy volunteers and regular blood donors. Available control metadata included age, sex, smoking status, and relevant comorbidities. Smoking status was available for 10 of 12 controls, of whom two reported current smoking and two had arterial hypertension. No active malignant or clinically apparent inflammatory disease was reported. Written informed consent was obtained from all participants prior to blood collection.

*sEV isolation and characterization.* Peripheral blood (5–15 mL) was collected in ethylenediaminetetraacetic acid (EDTA) tubes (#8076.3, Carl Roth, Karlsruhe-Mühlburg, Germany) from both controls and cancer patients. Samples were processed within 2 h to minimize plasma degradation. Plasma was obtained by centrifugation at 1200× *g* for 15 min, followed by two additional clearing spins at 1500× *g* for 15 min and 14,000× *g* for 35 min to remove cells, debris and large EVs. The 14,000× *g* supernatant was subjected to a 0.2 µm filtration step to further deplete larger vesicles and residual cell fragments. The filtrate was then ultracentrifuged at 100,000× *g* for 2 h to pellet sEVs, followed by one washing step in PBS with a second ultracentrifugation at 100,000× *g* for 2 h. The final sEV pellets were resuspended in Phosphate Buffered Saline (PBS, AC-BS-0002, Anprotec, Bruckberg, Germany), and total protein content was determined using a Lowry assay [19]. For downstream flow cytometric profiling with the MACSPlex EV Kit IO, a defined amount of vesicle protein (µg per reaction) was used as a surrogate measure of sEV input to ensure comparable vesicle loading across all samples. Particle-number-based normalization was not performed, as systematic *Nanoparticle tracking analysis* (NTA) measurements were not available for all samples.

NTA of sEVs was performed using a ZetaView PMX-120 instrument (PMX 120 ZetaView^®^ Mono Laser, Particle Metrix, Inning am Ammersee, Germany) equipped with a 640 nm laser and a CMOS camera (640 × 480 pixels, Particle Metrix). Samples were diluted in PBS to achieve a measurement range of approximately 50–400 particles per frame. NTA was performed on a subset of control sEV preparations (*N* = 5). For each preparation, videos were recorded at 11 cell positions at 25 °C, with camera sensitivity settings between 80 and 83. Data were processed and analyzed using ZetaView software (version 8.02.31, ZetaView^®^ Inning am Ammersee, Germany), yielding particle size distributions and particle concentrations (particles/mL) for each sample.

*Western blot analysis.* Protein expression in human plasma and sEV preparations was examined by immunoblotting. Briefly, equal amounts of protein (17 µg per lane) were separated by Sodium Dodecyl Sulfate (SDS)-PAGE (#0183.1, Carl Roth, Karlsruhe-Mühlburg, Germany) and transferred onto nitrocellulose membranes. Membranes were blocked in TBST (137 mM NaCl (#3957.2, Carl Roth, Karlsruhe-Mühlburg, Germany), 20 mM Tris (#10376743, Fisher Scientific, Waltham, MA, USA), pH 7.6, 0.1% [*v*/*v*] Tween-20 (#A4974,0250, Darmstadt, Germany)) and subsequently incubated overnight at 4 °C with primary antibodies against α-ACTININ-4 (sc-390205, SantaCruz, CA, USA), APOA1 (sc-376818, SantaCruz, CA, USA) and TSG101 (sc-7964, Lot#B2425, SantaCruz, CA, USA). After three washes in TBST (3 × 5 min), membranes were incubated with an HRP-conjugated secondary antibody (#7076, Cell Signaling Technology, Danvers, MA, USA) at room temperature, followed by another series of TBST washing steps (3 × 5 min). Bands were visualized using ECL Prime chemiluminescent substrate (GE Healthcare, Chicago, IL, USA) and documented with a LAS-4000 imaging system (Fujifilm, Düsseldorf, Germany). Ponceau S (#A2935,0500, Th. Geyer, Renningen, Germany) staining of the membranes was routinely performed as a loading control. Original blots are shown in Supplementary Figure S1.

*Flow cytometry.* Surface marker profiling of sEVs was performed using the MACSPlex EV Kit IO, human (order no. 130-122-209/130-108-813, Miltenyi Biotec, Bergisch Gladbach, Germany), which allows simultaneous flow-cytometric detection of 37 sEV surface epitopes and two isotype controls via fluorescently barcoded capture beads. For each sample, 15 µL of MACSPlex EV IO Capture Beads were incubated with 120 µL of sEV suspension under gentle agitation. sEVs bound to bead populations were subsequently stained with a cocktail of the three EV IO Detection Reagents CD9, CD63, and CD81 (5 µL each per reaction; total 15 µL) according to the manufacturer’s short protocol for tube or filter-plate format. After a 1 h incubation at room temperature, beads were washed twice with MACSPlex Buffer (3000× *g* for 5 min in tube format or vacuum/centrifugation for filter plates) and finally resuspended in buffer for acquisition. Flow cytometric measurements were carried out on a FACSCanto II flow cytometer (BD Biosciences, Franklin Lakes, NJ, USA) equipped with 488 nm and 633 nm lasers. Data were recorded with FACSDiva software (version 9.0.1, BD Biosciences, Franklin Lakes, NJ, USA)) and further processed using FlowJo software (version 10.8.1; BD Biosciences, Franklin Lakes, NJ, USA)). Vesicle-bound MACSPlex capture beads were first identified in forward- and side-scatter plots and gated as a distinct low-scatter bead population. Within this gate, the individual MACSPlex bead subsets corresponding to the different surface epitopes were resolved on FITC- versus PE-dot plots as discrete bead clusters. Each bead population was manually gated in FlowJo, and APC fluorescence intensity, reporting binding of the EV detection cocktail or APC-conjugated antibodies, were extracted for each epitope. For every sample, a buffer-only negative control was acquired in parallel, and its median APC signal was subtracted from the corresponding epitope-specific median fluorescence intensity to correct for non-specific background.

*Statistical and bioinformatic analysis.* Descriptive statistics (e.g., counts, proportions, medians and ranges) were obtained using Microsoft Excel 2016 (Microsoft Corporation, Redmond, WA, USA), which was sufficient given the primarily exploratory nature and limited sample size of the dataset. All subsequent inferential and multivariate analyses were performed in MetaboAnalyst 6.0. Raw flow cytometry output and clinical variables were first curated in Microsoft Excel 2016, including basic data cleaning (consistency checks, exclusion of technically invalid measurements) and extraction of clinical information from project registries and hospital information systems. The resulting dataset was then imported into MetaboAnalyst 6.0 [20] for statistical and multivariate analyses.

Concerning subgroup analysis of NLC and PDAC samples: The primary PDAC versus control analysis was performed within one experimental batch comprising 22 PDAC and 12 control samples. Therefore, this analysis did not require batch-based marker exclusion. For the PDAC versus NLC comparison, samples originated from two independent experimental batches: the PDAC/control batch described above and an additional NLC/control batch comprising 18 NLC and 6 control samples. To assess and mitigate batch effects, healthy control samples, present in both batches, were used as bridging controls. Based on these controls, four clear outlier control samples were excluded, and four batch-sensitive markers (CD3, CD56, HLA-ABC, MCSP) were removed before comparing PDAC and NLC samples. After this procedure, the remaining control samples no longer showed batch-driven clustering in principal component analysis (PCA) or heatmap analysis, supporting that major batch-related variation has been reduced. We therefore proceeded to compare PDAC and NLC samples using the reduced marker panel, while acknowledging that residual batch effects cannot be fully excluded.

All statistical and multivariate analyses were performed using MetaboAnalyst, version 6.0 (Statistics module; www.metaboanalyst.ca [20]). Data were log-transformed and Pareto scaled prior to analysis. Group separation between PDAC and controls was assessed by PCA, including evaluation of model significance by PERMANOVA with 999 permutations. PERMANOVA was used as an exploratory global test of group-associated differences in multivariate marker composition. Given the limited sample size and potential differences in within-group dispersion, PERMANOVA results were interpreted cautiously and were not used as evidence of diagnostic classification performance. Differential expression of individual sEV surface markers was examined using *t*-tests (PDAC versus controls; PDAC versus NLC). Resulting *p*-values were adjusted for multiple testing using the false discovery rate (FDR) approach. Volcano plots were generated from log_2_ fold-changes and FDR-corrected *p*-values, using the default FDR threshold of 0.1 in MetaboAnalyst. However, all prominently upregulated markers highlighted in the manuscript remained significant when a more stringent FDR cut-off of 0.05 was applied. The interpretation therefore focuses primarily on this more conservative marker set. Hierarchical clustering and heatmaps were created using Euclidean distance and Ward’s linkage on autoscaled marker intensities. For supervised classification, a Random Forest algorithm (ntree = 500, mtry = 7) was applied to discriminate PDAC from controls or NLC, reporting out-of-bag (OOB) error rates, confusion matrices and mean decrease in accuracy as a measure of variable importance. No nested cross-validation or additional model sensitivity analyses were performed. Therefore, OOB error rates were interpreted descriptively as exploratory internal performance estimates rather than as evidence of generalizable predictive accuracy. In addition, self-organizing maps (SOM; 1 × 3 grid, linear initialization, Gaussian neighborhood) were used as an unsupervised approach to visualize intrinsic cluster structure in the marker space and to explore concordance between SOM-derived clusters and clinical group labels. The manuscript underwent language editing and stylistic fine-tuning using ChatGPT with the GPT-5.4 model (OpenAI, San Francisco, CA, USA).

## 3. Results

### 3.1. Clinical Characteristics and Characterization of sEVs

Overall, we included 22 PDAC as well as 18 NLC patients and twelve healthy controls into our analysis. Clinical baseline characteristics on participants cohorts are listed in Table 1.

**Figure 1:**
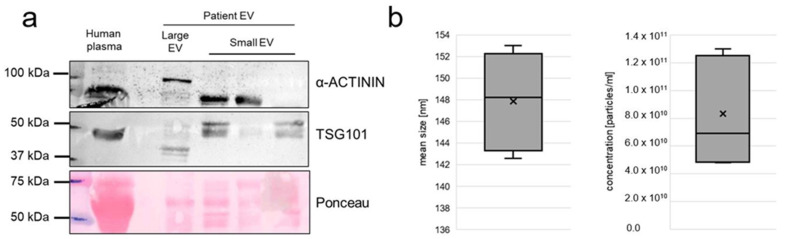
Characterization of plasma-derived small extracellular vesicles (sEV). (**a**) Representative Western blot of sEV preparations probed for canonical EV-associated TSG101 and negative markers for cellular contamination, confirming successful isolation of sEV. (**b**) Nanoparticle tracking analysis (NTA) performed on a subset of control sEV preparations (*N* = 5) demonstrates a particle size distribution in the sEV range. The median particle diameter was 148.2 nm (IQR 143.00–152.25 nm, left panel) and the median particle concentration of 6.90 × 10^10^ particles/mL (IQR 4.85 × 10^10^–1.25 × 10^11^ particles/mL (right panel; boxplots show median, interquartile range, whiskers and mean as cross) mean particle diameter and a mean particle concentration of.

Before immunophenotyping sEVs, we first confirmed successful enrichment of sEVs from plasma. In Western Blot analysis, sEVs from PDAC patients showed no expression of α-actinin as a surrogate marker for large EVs. Meanwhile, TSG101, a canonical marker of endosomal origin, was readily detected in all sEV samples (Figure 1a). Additional immunoblotting for ApoA-1 was performed to assess potential co-isolated lipoprotein-associated contaminants. This analysis did not indicate prominent enrichment of ApoA-1-positive contaminants in the sEV-enriched preparations. This reflects an effective reduction in major lipoprotein and cell-associated contaminants during the enrichment workflow (Supplementary Figure S2). Next, NTA was performed on sEV preparations from five healthy controls (*N* = 5; Figure 1b). The median particle diameter was 148.2 nm (Interquartile Range (IQR) 143.0–152.25 nm), consistent with enrichment of particles in the sEV size range. The median particle concentration was 6.90 × 10^10^ particles/mL (IQR 4.85 × 10^10^–1.25 × 10^11^ particles/mL), indicating reproducible recovery of particle-containing preparations suitable for downstream immunophenotypic profiling. In summary, this characterization demonstrates that our protocol yields a population of particles with biochemical and biophysical properties that are characteristic of sEVs and therefore suitable for downstream immunophenotypic profiling.

### 3.2. sEV from Pancreatic Ductal Adenocarcinoma Patients Display a Distinct Immunophenotypic Profile

**Figure 2:**
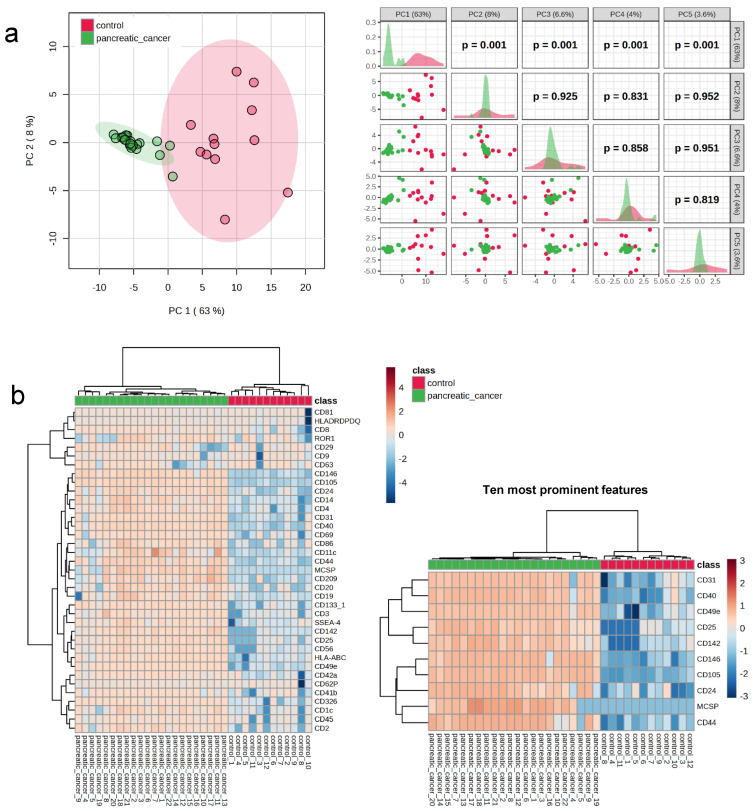
Immunophenotypic profiling of small extracellular vesicles (sEV) reveals a pancreatic cancer-specific vesiculome signature in peripheral blood. (**a**) Principal component analysis (PCA) based on immunooncology marker intensities shows an apparent group-associated shift between patients with pancreatic cancer (green) and healthy controls (red), mainly along PC1 (63% of variance), while control samples display heterogeneity, particularly along PC2, and several samples occupy partially overlapping regions. Group centroids with 95% confidence ellipses are shown. The global difference in vesicle surface marker composition between groups was supported by PERMANOVA (F = 89.8, R^2^ = 0.74, *p* = 0.001 based on 999 permutations), which was interpreted as an exploratory multivariate test rather than as evidence of complete separation. Scatterplot matrix of the first five principal components with overlaid kernel density estimates of the distribution of scores for each group; *p*-values indicate the significance of between-group differences for individual PCs, highlighting that PC1–PC3, but not higher components. PCA axes represent principal component scores derived from log-transformed and Pareto-scaled MACSPlex marker intensities. (**b**) Hierarchically clustered heatmap (Euclidean distance, Ward linkage) of standardized marker expression across all samples shows distinct clustering of pancreatic cancer versus control sEV profiles, with a subset of immunoregulatory and tumor-associated epitopes being prominently up- or downregulated in the cancer group. Heatmap colors represent standardized marker expression after log transformation and Pareto scaling.

PDAC is a systemic disease, facilitating significant changes in the immune cell function of patients [21,22]. Given their role as known mediators in both local and distant cell-cell communication [23], sEV may reflect these disease-associated immunological changes and provide insights into the current immune status of patients. We therefore aimed to characterize the immune profile of sEVs by comparing PDAC samples to healthy controls. By immunophenotyping plasma-derived sEV, we observed a distinct PDAC-associated signature compared with healthy controls. PCA, based on the MACSPlex immuno-oncology panel, showed an apparent group-associated shift between PDAC patients and controls, primarily along the first principal component. However, the separation between PDAC and control samples was not complete; control samples showed considerable heterogeneity. Particularly, along PC2, a small subset of PDAC samples clustered near the control group. These observations indicate that disease status contributed substantially to the variance in the dataset, while also underscoring relevant inter-individual variability within the control cohort. PERMANOVA supported an overall difference in multivariate marker composition between groups (F = 89.8, R^2^ = 0.74, *p* = 0.001 based on 999 permutations). This results were interpreted as an exploratory global test of group associated differences rather than as evidence of complete group separation or diagnostic classification (Figure 2a).

We next used heat mapping to visualize group-specific expression patterns at the level of individual epitopes. PDAC sEV consistently displayed increased signals for several immune and endothelial markers, including CD105, CD25, CD40, CD146, CD142, MCSP, CD24, CD44, CD31, CD20 and HLA-ABC, whereas the same epitopes appeared comparatively weak or even downregulated in healthy controls. The focused heatmap of the ten most discriminative features highlights that especially CD105, CD25, CD142, CD40, CD146, MCSP, CD24 and CD31 contribute strongly to the observed group separation, pointing to a cancer-associated remodeling of the circulating sEV surface proteome (Figure 2b). Furthermore, heatmap analysis demonstrates the expression pattern of the canonical pan-EV markers CD9, CD63 and CD81. These tetraspanins do not show a systematic over- or underexpression in either group, but rather comparable signal intensities across PDAC and control samples. This similarity in CD9/CD63/CD81 levels argues against major differences in overall sEV capture efficiency or gross vesicle input between the two cohorts. This supports the assumption that the pronounced shifts seen for other epitopes are unlikely to be driven by technical bias alone.

**Figure 3:**
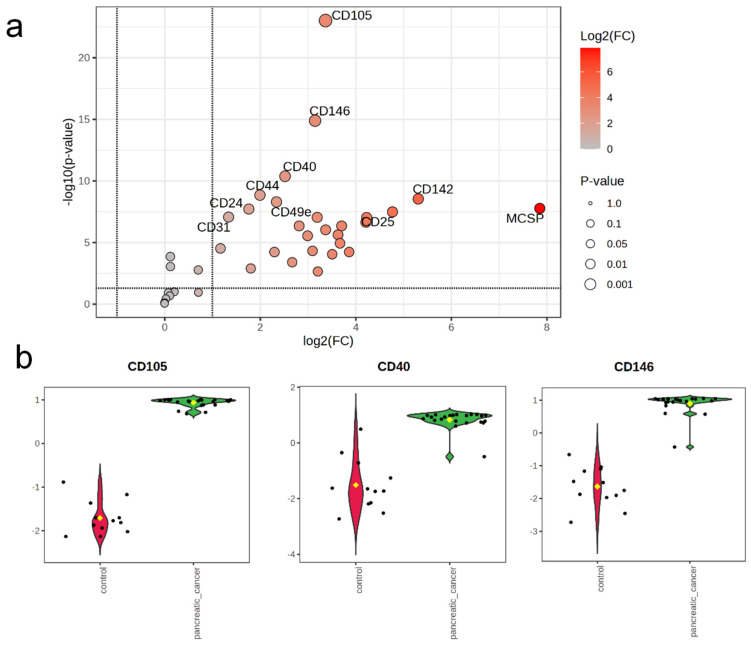
Differential expression of sEV surface markers identifies a subset of strongly upregulated immunophenotypic features in pancreatic cancer. (**a**) Volcano plot displaying log_2_ fold changes (log_2_(FC), pancreatic cancer vs. control; x-axis) against −log10(*p*-values) from unpaired tests (y-axis) for all analyzed EV immuno-oncology markers. A false discovery rate (FDR) threshold of 0.1 was applied (horizontal and vertical dotted lines), and the ten most significant markers are labeled, highlighting pronounced overexpression of MCSP, CD105, CD40, CD146, CD142, CD25 and others in pancreatic cancer–derived sEV. (**b**) Violin plots of normalized expression values for the three most significant markers (CD105, CD40, CD146) illustrate the distribution and group medians of marker intensities in controls versus pancreatic cancer patients, confirming marked and consistent upregulation of these epitopes on circulating sEV in the cancer group. Violin plots show normalized MACSPlex fluorescence intensities after background subtraction and preprocessing.

Differential marker analysis of the data identified a subset of sEV surface epitopes that were significantly enriched in pancreatic cancer patients compared with healthy controls. The volcano plot (Figure 3a) summarizes these changes by plotting log_2_ fold changes against the corresponding −log_10_(*p*-values), with an FRD threshold of 0.1 applied to account for multiple testing. However, the key upregulated markers discussed below remained significant at a more stringent FDR threshold of 0.05. Among the identified epitopes, CD105 displayed the most pronounced alteration, exhibiting both the highest statistical significance (−log_10_ *p*-value) and a substantial increase in signal intensity on pancreatic cancer-derived sEV. MCSP, CD142 and CD25 also showed large effect sizes with high fold changes, underscoring marked differences in their vesicular presentation between the two groups. Additional significantly upregulated markers include CD40 and CD146, as well as several other immune- and endothelium-associated antigens, which together define a coordinated, cancer-associated remodeling of the sEV surface proteome.

To illustrate the magnitude and consistency of these changes at the individual level, violin and box plots for selected epitopes (CD105, CD40, CD146) depict a clear right shift and broader distribution of signal intensities in the PDAC group compared with controls (Figure 3b). This pattern indicates that the upregulation of these markers is not driven by single outliers but represents a recurrent feature across most patients, supporting their potential utility as circulating sEV-based biomarkers for pancreatic cancer. Fold changes in all significantly expressed epitopes are listed in Table 2. To further explore the diagnostic potential of selected candidate markers, we performed an exploratory ROC analysis for the most prominent altered sEV surface epitopes. These analyses were restricted to individual markers and interpreted descriptively, given the limited sample size and lack of an independent validation cohort. CD105 showed an AUC of 1.00 (95% CI 1.00;1.00), CD146 an AUC of 1.00 (95% CI 1.00;1.00), and CD40 an AUC of 0.99 (95% CI 0.97; 1.00) for distinguishing PDAC patients from healthy controls (Supplementary Figure S3).

### 3.3. Exploratory Machine-Learning Analysis Supports a PDAC-Associated sEV Immunophenotype

**Figure 4:**
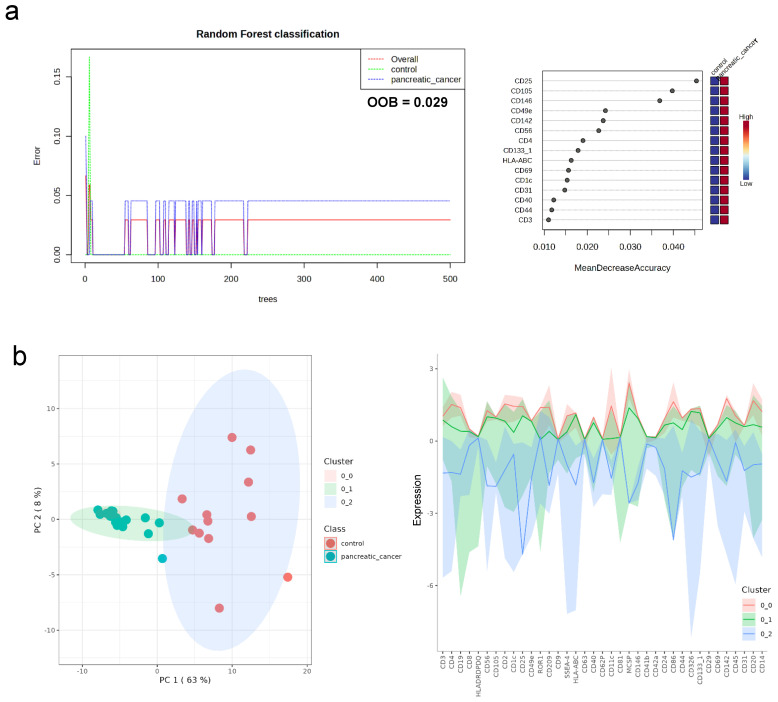
Exploratory supervised and unsupervised machine-learning analyses of pancreatic cancer and control small extracellular vesicles (sEV) immunophenotypes. (**a**) Random Forest classification using the full panel of normalized sEV immunooncology markers (ntree = 500, mtry = 7) shows a rapid decrease and early stabilization of the out-of-bag (OOB) error, with a final OOB error rate of 0.029. The confusion matrix indicates correct classification of all controls (12/12) and only a single misclassified pancreatic cancer sample (21/22 correctly classified), corresponding to a class error of 0.05 for the cancer group. The variable importance plot (MeanDecreaseAccuracy) identifies a compact set of surface markers contributing most strongly to exploratory classification within this dataset. Variable importance is shown as the mean decrease in accuracy. (**b**) Self-organizing map (SOM) analysis of the same normalized marker data (1 × 3 grid, Gaussian neighborhood, linear initialization) provides an unsupervised assessment of the intrinsic structure of the marker data. In the SOM-projected PCA score plot, samples group into distinct clusters that almost completely correspond to clinical class labels (controls versus pancreatic cancer), and the cluster-wise marker profiles show coherent, disease-associated shifts across the measured epitopes.

We next asked whether these distinct patterns of sEV immune profiles could be potentially used in a diagnostic context. Supervised and unsupervised machine-learning approaches were used to explore whether the observed sEV marker patterns were sufficient to separate PDAC patients and controls within this dataset. Given the limited sample size and absence of an independent validation cohort, these analyses were interpreted as exploratory internal consistency analyses rather than as evidence of diagnostic model performance. In the Random Forest classifier all control samples (12/12) and 21 of 22 PDAC samples were correctly assigned to their respective groups, corresponding to a total out-of-bag error rate of only 2.9% (Figure 4a).

The most informative Random Forest features, including CD105, CD25, CD142 and CD40, overlapped with the marker set highlighted by differential expression analysis and heatmap visualization, which also included CD146 and MCSP. This overlap supports the internal consistency of the observed sEV immunophenotypic pattern across complementary analytical approaches, while not constituting external validation. SOM analysis of the same normalized marker matrix provided an unsupervised assessment of the intrinsic marker structure. The SOM was constructed without using class labels and therefore reflects only the intrinsic structure of the marker data. Yet, the three SOM-derived clusters almost perfectly recapitulated the clinical groups, with one cluster predominantly containing controls and the remaining clusters comprising pancreatic cancer patients (Figure 4b). The corresponding SOM profile plot showed coherent, disease-associated shifts in marker expression across the measured epitopes within each cluster. Notably, markers contributing to the Random Forest analysis also showed pronounced cluster-specific expression patterns in the SOM. This suggests that the supervised and unsupervised approaches captured overlapping aspects of the same sEV immunophenotypic structure.

### 3.4. Exploratory Longitudinal Shifts of Circulating sEV Immunophenotypes During Chemotherapy

**Figure 5:**
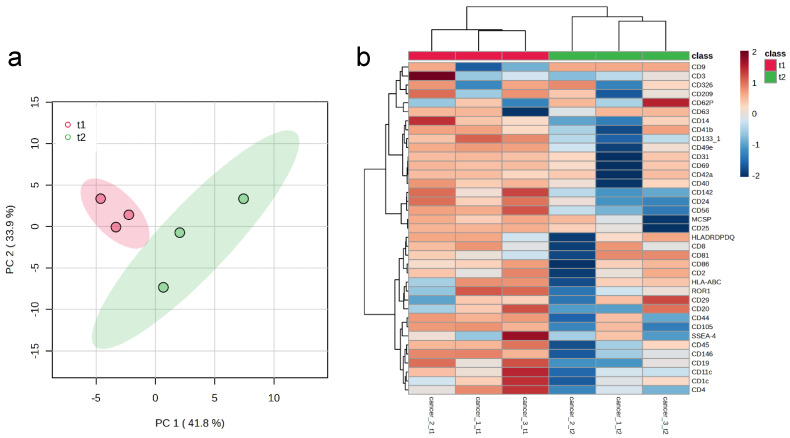
Exploratory longitudinal analysis of small extracellular vesicles’ (sEV) surface marker profiles in three pancreatic cancer patients: (**a**) Principal component analysis (PCA) based on normalized immunooncology marker intensities for paired baseline (t1, red) and 3 months follow up samples (t2, green) from three patients illustrates an observable shift of the vesiculome profile over time at the individual level, with t1 and t2 samples occupying distinct regions in PC space. PCA axes represent principal component scores derived from log-transformed and Pareto-scaled MACSPlex marker intensities. (**b**) Hierarchically clustered heatmap of standardized marker expression (Euclidean distance, Ward linkage, autoscaled features) for the same six samples shows that all t1 samples cluster together and are separated from the corresponding t2 samples, which display a coherent change in multiple immune and endothelial markers. Heatmap colors represent standardized marker expression after log-transformation and Pareto-scaling. Given the very small sample size, these longitudinal observations must be regarded as strictly exploratory and hypothesis-generating only; they suggest, but do not prove, that the sEV immunophenotype may dynamically reflect disease course or treatment effects in pancreatic cancer.

PDAC samples were derived from chemotherapy-naïve patients. To assess whether changes in sEV immune profile are associated with disease activity, we performed an exploratory longitudinal analysis of sEV surface marker profiles in a small subset of pancreatic cancer patients with available follow-up samples. For four patients, a second blood draw was obtained approximately three months after baseline and after initiation of systemic chemotherapy. One patient had to be excluded from the longitudinal analysis because raw flow cytometry data showed insufficient sEV event counts and thus represented a technical outlier. In the remaining three patients, PCA based on normalized marker intensities suggested a noticeable shift of the vesicular immune profile between baseline and follow-up at the individual level, with t1 and t2 samples occupying distinct regions in PC space (Figure 5a). Hierarchical clustering and heatmap visualization of the same markers supported these observations: all t1 samples clustered together and were separated from the corresponding t2 samples, which displayed a coherent change in multiple immune- and endothelium-associated epitopes (Figure 5b).

**Figure 6:**
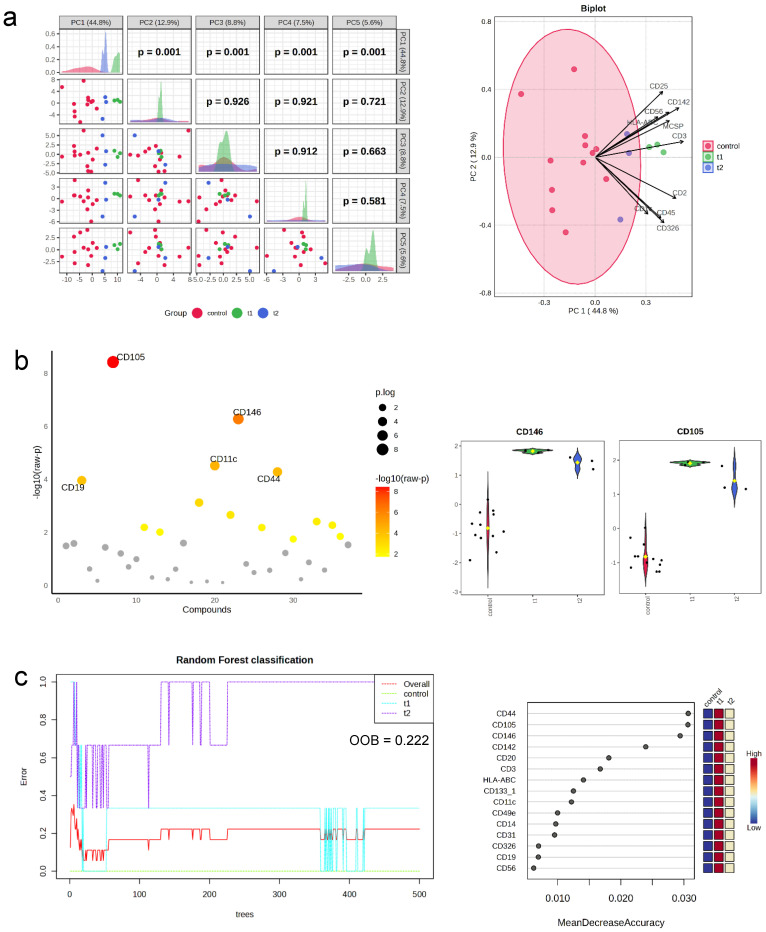
Exploratory three-class analysis of circulating small extracellular vesicles’ (sEV) immunophenotypes in controls and pancreatic cancer patients at baseline and follow-up. (**a**) Principal component analysis (PCA) comparing healthy controls (control, red), patients at diagnosis (t1, green), and the same patients after approximately three months of chemotherapy (t2, blue). The pairwise PC plots and the PCA biplot demonstrate an observable separation of cancer samples from controls along PC1 and PC2, with PERMANOVA confirming overall group differences (F = 10.874, R^2^ = 0.592, *p* = 0.001 based on 999 permutations). Loading vectors indicate that several angiogenesis and immune-related markers contribute most strongly to the displacement of t1/t2 samples away from the control cluster. PCA axes represent principal component scores derived from log-transformed and Pareto-scaled MACSPlex marker intensities. (**b**) Results of one-way ANOVA across the three groups (control, t1, t2) displayed as a bubble plot, highlighting CD105, CD146, CD11c, CD44 and CD19 as the most significantly differentially expressed markers. Representative violin plots for CD146 and CD105 illustrate markedly increased sEV-associated expression at t1 relative to controls and persistently elevated levels at t2, suggesting an enduring pro-angiogenic and immune-activated vesicle phenotype despite chemotherapy. (**c**) Random Forest classification using the same marker panel to distinguish control, t1 and t2 samples. The left panel shows the out-of-bag (OOB) error rate over 500 trees, with a final overall OOB error of 0.222 and correct classification of all 12 control samples, whereas most misclassifications occur between t1 and t2. The right panel depicts variable importance (mean decrease in accuracy), again identifying key contributors to group separation. Variable importance is shown as the mean decrease in accuracy. Taken together, these descriptive analyses illustrate measurable temporal variation in sEV surface marker profiles in three paired PDAC samples. However, given the very small number of longitudinal samples and heterogeneous treatments, all findings in this figure must be considered strictly exploratory and hypothesis-generating.

In an exploratory three-class analysis, we next compared the sEV immunophenotype of healthy controls (*N* = 12) with baseline (t1, *N* = 3) and three-month follow-up samples (t2, *N* = 3) from pancreatic cancer patients. PCA revealed an observable separation of cancer samples from controls along the first principal component, while t1 and t2 samples occupied neighboring but distinct regions in PC space. This suggests that the vesicular surface signature of pancreatic cancer is already strongly altered at diagnosis and undergoes further, though more subtle, modulation during chemotherapy (Figure 6a). Univariate statistics highlighted several angiogenesis- and immune-related markers, including CD105 and CD146, as being markedly upregulated in both t1 and t2 compared with controls, with persistently high levels at follow-up rather than normalization (Figure 6b).

To assess whether these patterns were sufficient to discriminate the three groups, we trained a Random Forest classifier on the same marker set. The model correctly classified all control samples and most cancer samples, yielding an overall OOB error rate of 0.222, with misclassifications occurring mainly between t1 and t2 rather than between controls and cancer. Variable importance analysis (mean decrease in accuracy) identified CD44, CD105, CD146, CD142 and related markers as the main contributors to three-class separation (Figure 6c), largely overlapping with the epitopes highlighted in the univariate analysis. Taken together, these descriptive longitudinal observations are compatible with measurable temporal variation in the sEV surface marker profiles of these three PDAC patients during the first months of systemic chemotherapy. However, due to the very small sample size, heterogeneous treatment regimens, and absence of outcome correlation, no statistical, biological, or clinical conclusions regarding treatment response, disease monitoring, or outcome prediction can be drawn. These observations therefore require confirmation in larger, prospectively collected longitudinal cohorts with predefined sampling time points.

### 3.5. Entity-Specific sEV Epitope Fingerprints Distinguish Pancreatic Cancer from NLC

**Figure 7:**
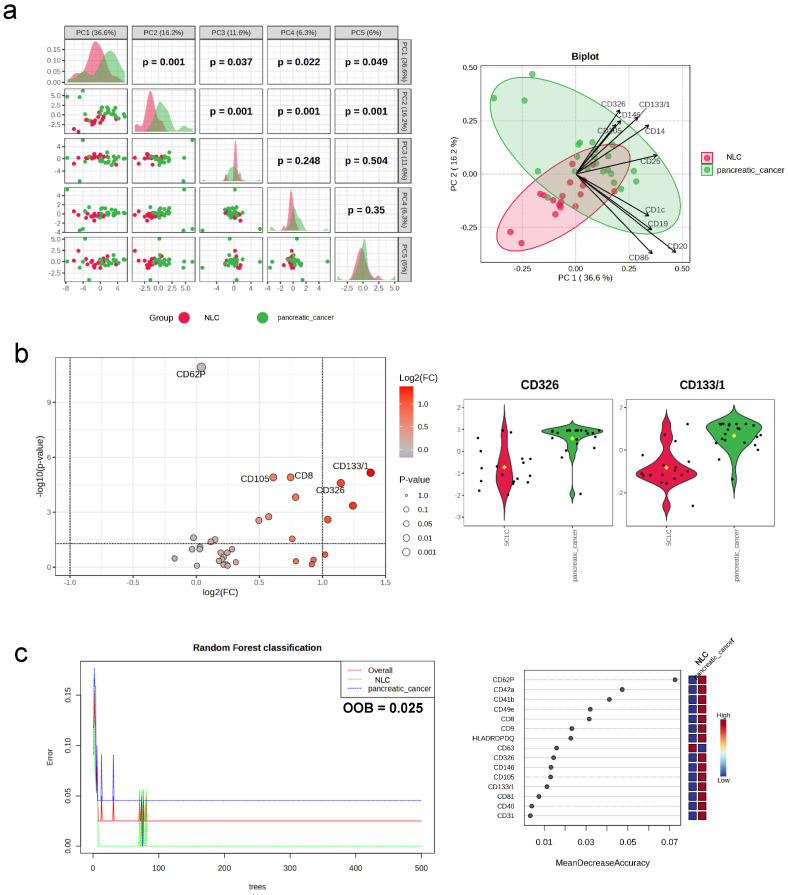
Immunophenotyping of small extracellular vesicles (sEV) distinguishes pancreatic cancer from neuroendocrine lung cancer (NLC). (**a**) Principal component analysis (PCA) shows that pancreatic cancer and NLC patients occupy partially overlapping but clearly shifted regions in PC space, with significant overall group separation (PERMANOVA (*p* = 0.001)). Pairwise PC plots and the PCA biplot indicate that several tumor- and epithelium-associated markers, as well as myeloid and T-cell-related epitopes, contribute most strongly to the displacement of pancreatic cancer samples away from the NLC cluster. PCA axes represent principal component scores derived from log-transformed and Pareto-scaled MACSPlex marker intensities. (**b**) Univariate comparison of sEV surface markers between pancreatic cancer and NLC. The volcano plot and FDR-corrected significance plot highlight CD326 (EpCAM) and CD133/1 as the most strongly enriched markers on pancreatic cancer–derived sEV, whereas platelet-associated CD62P is markedly higher in NLC. Representative violin plots for CD326 and CD133/1 illustrate consistently elevated vesicular expression in pancreatic cancer compared with NLC across individual patients, in line with the known epithelial and stem-like phenotype of pancreatic ductal adenocarcinoma. Violin plots show normalized MACSPlex fluorescence intensities after background subtraction and preprocessing. (**c**) Exploratory supervised Random Forest classification separates pancreatic cancer and NLC within this dataset with an out-of-bag error rate of 0.025 and a correct assignment of 18/18 NLC and 21/22 PDAC samples. Variable importance is shown as the mean decrease in accuracy. The variable importance plot (mean decrease in accuracy) key contributors to classification performance, suggesting that differences in platelet-derived vesicles and epithelial/tumor-associated sEV may contribute to the observed separation between pancreatic cancer and NLC.

While our observations are promising, our findings do not confirm whether the immune profile observed reflects general changes in cancer patients or a specific profile inherently associated with PDAC (entity-specific immune profile). We chose NLC as the second cancer entity for analysis. This choice was made as NLC is also an aggressive, systemic disease [24]—similar to PDAC. Immunophenotyping of circulating sEV also revealed distinct vesicle signatures for PDAC and NLC. In PCA, samples from PDAC (*N* = 22) and NLC patients (*N* = 18) occupied partly overlapping but clearly shifted regions in PC1–PC2 space, with significant overall group separation confirmed by PERMANOVA (*p* = 0.001). The PCA biplot indicated that epithelial and stemness-associated markers such as CD326 (EpCAM) and CD133/1, together with several myeloid and endothelial epitopes (CD11c, CD14, CD105, CD146), drove the displacement of pancreatic cancer samples, whereas NLC samples aligned more closely with vectors for platelet-related markers (Figure 7a). Univariate analyses supported these multivariate findings. The volcano plot and FDR-corrected significance plot highlighted CD326 and CD133/1 as the most strongly enriched markers on pancreatic cancer-derived sEV, while CD62P stood out as the top marker increased in NLC. Violin plots for CD326 and CD133/1 illustrated consistently higher sEV-associated expression in pancreatic cancer compared with NLC, consistent with the epithelial and stem-like phenotype of PDAC (Figure 7b).

To explore whether these differences were sufficient to separate entities within this dataset, we trained a Random Forest classifier using the same marker set. The model achieved an OOB error rate of 0.025, correctly assigning 21/22 pancreatic cancer and all NLC samples to their respective classes. Variable importance analysis identified CD62P, CD41b and CD42a as the strongest contributors to classification, followed by CD146, CD326, CD105, CD31 and CD133/1, indicating that both platelet-derived vesicles and epithelial/tumor-associated sEV jointly underpin the separation between entities (Figure 7c). Although these data are derived from a single, moderately sized cohort and must therefore be regarded as exploratory, they suggest that tumor entity-specific sEV surface marker fingerprints could, in principle, support non-invasive differentiation between PDAC and NLC.

## 4. Discussion

The primary aim of our study was to evaluate the potential of sEVs isolated from peripheral human blood as a liquid biopsy approach, thereby avoiding more invasive sampling strategies than those used in previous studies investigating sEVs [16,17]. In accordance with the recently updated hallmarks of cancer [18], we hypothesized that PDAC, as a systemic disease, induces changes that are reflected in the immunoprofile of sEVs secreted by bystander immune cells. Notably, our exploratory study identified distinct sEV immunophenotypes in patients with PDAC compared with healthy controls. Exploratory machine learning analyses showed a high degree of separation between PDAC and control samples within the present dataset. However, given the small sample size and lack of an independent validation cohort, these findings should be interpreted as evidence of internal consistency rather than as validated diagnostic performance. Importantly, this discrimination was driven by a composite marker pattern rather than by individual markers, supporting the concept that circulating sEV reflect systemic tumor associated processes rather than isolated molecular alterations.

A closer examination of the epitopes with higher expression in PDAC samples revealed several sEV-associated markers that can be grouped according to established biological associations, while more speculative interpretations regarding their cellular origin and functional consequences require caution. These included angiogenesis-associated markers (CD105, CD146, CD31 [25,26,27]), immune-regulatory markers (CD40, CD25 [28,29]), coagulation-related proteins (CD142 [30]), and markers linked to invasion and cellular plasticity (MCSP, CD24 [31,32]). In particular, CD25 is a marker of regulatory T-cell activity [33] and therefore supports the presence of an EV-associated signature of immune suppression, as a hallmark of PDAC. CD40, a member of the tumor necrosis factor receptor superfamily expressed on both immune and tumor cells [34,35], may reflect tumor immune interactions. Regarding the angiogenic component of PDAC, the high expression of CD105 (endoglin) on sEV is of particular interest. CD105 is upregulated in proliferating endothelium [36], further underscoring the pronounced angiogenic component of the observed sEV phenotype. Taken together, these findings suggest that sEVs function as an integrated, system-level readout of tumor–host interactions rather than as passive carriers of tumor-derived material. This interpretation is supported by a previous small study investigating the sEV proteome in patients with advanced PDAC, in which pathway analysis indicated strong involvement of the sEV proteome in immune response, thrombosis, metastasis, and proliferation [37]. Overall, these data are consistent with the concept that sEV capture central aspects of tumor biology, including vascular remodeling, immune suppression, cancer-associated thrombosis, and tumor cell plasticity [18]. However, the present study cannot determine the precise cellular origin or functional consequences of these vesicles associated epitopes, and the observed sEV immunophenotype should therefore be interpreted as a candidate systemic readout of PDAC associated biology rather than as mechanistic evidence.

Although these observations are consistent with the concept that PDAC is a systemic disease [38], we additionally sought to compare PDAC with another aggressive malignancy characterized by early metastasis and paraneoplastic phenomena, namely NLC. This comparison was intended to clarify whether the immunoprofile observed in PDAC is specific to this tumor entity or rather reflects a more general profile associated with highly aggressive cancers. Comparative analyses across tumor entities demonstrated that PDAC and NLC exhibit distinct immunoprofile fingerprints, suggesting that sEV immunoprofiles encode disease-specific information. Although individual markers were not unique to PDAC, differences in their relative abundance and combinatorial patterns supported exploratory separation of PDAC and NLC within the present dataset. These findings support the concept that specificity in EV-based liquid biopsy arises from multidimensional pattern recognition rather than from the expression of single markers. In this context, sEV profiles may represent candidate entity-associated “fingerprints” that reflect both tumor identity and systemic disease characteristics.

Having established that patients with PDAC display a distinct sEV immunoprofile, we next asked whether this approach might also be suitable for monitoring disease burden. Initial studies have focused on specific sEV protein markers [39] or genomic DNA extracted from sEV [40]. Compared with these cargo-based or single-marker approaches, the present study focuses on multiplexed surface immunophenotyping of plasma-derived sEV-enriched preparations. This strategy does not aim to replace molecular cargo analyses but provides a complementary readout of vesicle-associated surface epitopes that may reflect tumor–host interactions, immune modulation, endothelial activation, and coagulation-related processes. In addition, whereas several previous EV-based PDAC biomarker studies used pancreatic juice or cyst fluid [15,17], our approach relies on peripheral blood plasma, which is more suitable for repeated sampling and longitudinal exploratory analyses. In contrast to studies focusing on preoperative samples [39], we aimed to assess a longer disease trajectory by analyzing the sEV immunoprofile three months after the initiation of chemotherapy. Although our data are clearly exploratory, the available longitudinal samples illustrate that paired sEV immunophenotyping is technically feasible and can capture measurable intra-individual temporal variation. In this small subset, follow-up samples did not converge toward the control group but remained visually separated from controls in multivariate analyses, suggesting that elements of the PDAC-associated sEV marker pattern were still detectable after initiation of chemotherapy. At the same time, baseline and follow-up samples showed observable differences from each other, indicating temporal variation within individual patients. However, given the very small number of paired samples, heterogeneous treatments, and lack of clinical outcome correlation, these observations do not allow conclusions regarding treatment response, persistent biological activity, disease monitoring, or early detection of resistance. They should therefore be interpreted strictly as descriptive feasibility observations that require evaluation in larger, prospectively sampled longitudinal cohorts.

At the same time, our data suggest that sEV profiles are not entirely static but may capture dynamic changes over time. Even within the limited number of longitudinal samples, follow-up profiles could be distinguished from baseline samples, indicating that the relative expression of key markers undergoes measurable shifts during treatment. These changes may reflect evolving tumor biology, treatment response, or alterations in tumor–host interactions. Although clearly exploratory, this observation points to an additional layer of biological information embedded in sEV profiles, with potential relevance for real-time disease monitoring and the early detection of treatment resistance.

Compared with established biomarkers, sEV-based profiling may offer complementary advantages. CA19-9, although widely used, is limited by reduced sensitivity and specificity, particularly in early-stage disease and in CA19-9-negative patients [41,42,43,44]. A substantial proportion of patients with PDAC do not exhibit elevated CA19-9 levels, partly because of absent Lewis antigen expression, which may result in false-negative findings [8]. In this context, alternative biomarkers are urgently needed. Previous studies have shown that exosomal CD40 alone achieves good diagnostic performance (AUC ~0.83), which can be further improved by combining CD40 with CD25 and CA19-9 (AUC up to 0.92 [45]). Our findings support such an integrative approach and suggest that sEV-based marker panels may complement or enhance current biomarker strategies.

These exploratory findings may have potential clinical implications if confirmed in larger validation cohorts. If validated, sEV profiling could (1) potentially contribute to the differentiation between malignant and benign pancreatic conditions. Furthermore, (2) this approach could be explored as a tool for longitudinal monitoring of disease activity and treatment response. Given their accessibility in peripheral blood and their ability to reflect both tumor-intrinsic and microenvironmental processes, sEVs represent a promising platform for minimally invasive disease assessment.

### Limitations

A major strength of our study is the use of a standardized, multi-step workflow for sEV isolation and immunophenotyping, combining differential ultracentrifugation, size filtration, Western blot-based marker assessment, nanoparticle tracking analysis, and bead-based capture using the MACSPlex EV Kit IO. This approach provides a standardized technical framework and an effective “double safeguard” to enrich for sEV and to interrogate vesicle-associated rather than freely circulating epitopes. Nevertheless, several important limitations should be acknowledged.

First, despite this multiparametric characterization, we did not perform a fully comprehensive, guideline-level sEV characterization, such as density-based separation, electron microscopy, or systematic nanoparticle tracking analysis across all samples. Vesicle purity and the precise contribution of different EV subtypes, therefore, cannot be definitively established. Although additional ApoA-1 immunoblotting was performed to assess major lipoprotein-associated contamination, we did not perform quantitative lipoprotein profiling or density-based separation to fully resolve EV from co-isolated plasma lipoproteins. Accordingly, our findings should be interpreted as relating to a sEV-enriched plasma fraction rather than to a rigorously isolated, homogeneous exosome population. In addition, MACSPlex input was normalized by total protein rather than by absolute particle number, such that differences in protein cargo density or residual co-isolated plasma proteins between groups may still have influenced apparent marker intensities. This protein-based normalization was used as a pragmatic surrogate to support comparable MACSPlex loading. However, NTA was performed only in representative control preparations and not systematically across all samples. Therefore, no NTA-informed sensitivity analysis using particle counts could be performed. Consequently, we cannot assess group-specific differences in absolute particle concentration or size distribution between PDAC patients and controls. Moreover, our isolation and profiling strategy was optimized for sEV-enriched preparations and therefore did not systematically assess larger EV subpopulations. This represents an important limitation, as larger EVs may also carry biologically and clinically relevant information in PDAC and other malignancies. The selective focus on sEV was chosen to enable standardized, multiplexed, and high-throughput-compatible surface marker profiling using a commercially available platform with established technical applicability to sEV-enriched samples. However, future studies should directly compare sEV and larger EV fractions using orthogonal isolation, imaging, and molecular profiling approaches to determine whether these vesicle populations provide complementary or distinct biomarker information.

Second, this was a single-center study with a modest and primarily exploratory sample size (22 patients with PDAC, 18 with NLC, and 12 healthy controls), which limits statistical power for the detection of more subtle effects and does not support definitive conclusions for smaller subgroups. The approximately 2:1 allocation of PDAC cases versus controls was intentionally chosen to facilitate biomarker discovery rather than based on formal power calculations. The PDAC versus NLC comparison was nearly balanced and therefore enabled an initial exploratory assessment of tumor entity-associated sEV patterns. The apparent discrimination performance observed within this cohort should therefore be interpreted with caution. Recruitment extended over a prolonged time during which standards of diagnostic work-up and systemic treatment for PDAC and NLC evolved, potentially introducing temporal heterogeneity that was not fully controlled for. Controls were recruited from hospital staff, volunteers, and blood donors and were not perfectly matched to patients with respect to age, comorbidities, or subclinical inflammation. Although available metadata did not indicate active malignant or clinically apparent inflammatory disease among controls, residual confounding by these factors therefore cannot be excluded. This heterogeneity was also reflected in the PCA (Figure 2a), where control samples did not form a compact cluster, particularly along PC2. Therefore, the observed multivariate group shift should be interpreted cautiously and may partly reflect heterogeneity within the control cohort in addition to PDAC-associated differences. Furthermore, longitudinal analyses were limited to three patients with PDAC who had paired baseline and post-chemotherapy samples and heterogeneous treatment regimens. These time course data should therefore be regarded as hypothesis generating rather than suitable for firm conclusions regarding dynamic biomarker behavior or outcome prediction. In addition, the NLC cohort included patients with different disease stages and treatment contexts at the time of sampling. Although most NLC samples were obtained before systemic therapy or during treatment-free intervals, treatment and stage-related effects on sEV release or marker expression cannot be fully excluded.

Third, from a statistical and bioinformatic perspective, the study benefits from complementary analytical strategies, including univariate testing, multivariate analysis, and machine learning-based classification, which together provide a coherent internal picture and support the internal consistency of the identified PDAC-associated sEV signature. However, all models were developed and evaluated within a single dataset without an independent validation cohort, and the risk of overfitting cannot be excluded, particularly given the high dimensionality of the marker panel relative to sample size. Accordingly, the machine learning analyses should be regarded as exploratory pattern recognition tools rather than as validated predictive models. Although the main highlighted markers remained significant at an FDR threshold of 0.05, the use of exploratory screening thresholds and the limited sample size still allows false positive findings, especially among markers with moderate effect sizes. Moreover, the MACSPlex EV Kit IO interrogates a fixed immunooncology surface marker panel, and we did not integrate orthogonal omics layers or established clinical biomarkers such as CA19-9 into the modeling framework. Consequently, the broader mechanistic context of the identified signature and its incremental value beyond standard clinical parameters remain to be defined. External validation in larger, prospectively collected, and clinically well-annotated cohorts, ideally with predefined endpoints such as treatment response and survival, will therefore be essential to confirm both the diagnostic promise and the potential prognostic utility of the proposed vesicle-based marker constellation.

To mitigate these limitations within the scope of the present exploratory dataset, we implemented several experimental and analytical safeguards. These included a standardized multi-step sEV enrichment workflow, orthogonal characterization by Western blotting and NTA in representative samples, assessment of canonical tetraspanin signals to evaluate gross differences in vesicle capture, and the use of complementary statistical approaches including FDR-corrected univariate testing, PCA/PERMANOVA, hierarchical clustering, Random Forest classification, and self-organizing maps. In the PDAC versus NLC comparison, potential batch effects were specifically addressed by identifying and removing batch-sensitive markers based on control samples present in both experimental batches. However, these measures can only strengthen internal consistency and reduce obvious technical bias. They cannot replace independent external validation, prospective sampling, or matched control cohorts. We therefore interpret the present findings as hypothesis-generating and as a rationale for future validation studies rather than as definitive evidence of clinical diagnostic performance.

## 5. Conclusions

This exploratory proof-of-principle study suggests that sEV immunophenotyping may capture biologically meaningful aspects of PDAC. The identified multidimensional marker patterns were consistent with key tumor-associated processes and, in a very small longitudinal subset, showed measurable temporal variation. These findings support further investigation of sEV as a potential liquid biopsy platform. Future studies are warranted to validate these observations in larger, independent cohorts to define their possible role in clinical decision-making.

## Figures and Tables

**Table 1:** Clinical baseline characteristics of participants.

Pancreatic Ductal Adenocarcinoma	
Male	13 (59.10%)
Female	9 (40.90%)
Age (min–max) years	63.23 (32–77)
Resectable	12 (54.55%)
Borderline resectable	7 (31.82%)
Locally advanced	3 (13.64%)
Palliative	0 (0%)
**Neuroendocrine Lung Cancer (Extensive Disease)**	
Male	11 (61.11%)
Female	7 (38.89%)
Age (min–max) years	64 (47–85)
Small cell lung cancer	17 (94.44%)
Large cell lung cancer	1 (5.56%)
Extensive disease	15/18 (83.33%)
Limited disease	3/18 (16.67%)
*Treatment status at sampling*	
chemotherapy-naïve or ≥7 days	17/18 (94.44%)
1 day after chemotherapy	1/18 (5.56%)
**Healthy Controls**	
Male	5 (41.67%)
Female	7 (58.33%)
Age (min–max)	49 (30–54)
Smoking	2/10 (20.00%)
Arterial hypertension	2/12 (16.67%)

**Table 2:** Fold changes and *p*-values of significantly expressed in epitopes comparing pancreatic cancer patients against healthy controls (volcano plot analysis).

	Fold Change	Log_2_(Fold Change)	Adjusted *p*-Value	−log10(*p*-Value)
**CD105**	10.312	3.366	9.6372 × 10^−24^	23.016
**CD146**	8.860	3.147	1.2934 × 10^−15^	14.888
**CD40**	5.731	2.519	4.1885 × 10^−11^	10.378
**CD44**	3.979	1.993	1.4143 × 10^−9^	8.850
**CD142**	39.597	5.307	2.8665 × 10^−9^	8.543
**CD49e**	5.062	2.340	5.0165 × 10^−9^	8.300
**MCSP**	230.770	7.850	1.683 × 10^−8^	7.774
**CD24**	3.389	1.761	1.9013 × 10^−8^	7.721
**CD25**	27.236	4.767	3.2449 × 10^−8^	7.489
**CD31**	2.525	1.336	8.5147 × 10^−8^	7.070
**CD14**	9.135	3.192	9.0143 × 10^−8^	7.045
**CD133_1**	18.714	4.226	9.1998 × 10^−8^	7.036
**CD56**	18.514	4.211	2.2644 × 10^−7^	6.645
**CD4**	13.022	3.703	4.4782 × 10^−7^	6.349
**CD209**	7.027	2.813	4.4782 × 10^−7^	6.349
**HLA-ABC**	10.323	3.368	9.2227 × 10^−7^	6.035
**CD326**	12.363	3.628	2.349 × 10^−6^	5.629
**CD11c**	7.946	2.990	2.8248 × 10^−6^	5.549
**CD3**	12.720	3.669	1.1445 × 10^−5^	4.941
**CD69**	2.245	1.167	2.9952 × 10^−5^	4.524
**CD2**	8.540	3.094	4.7547 × 10^−5^	4.323
**CD20**	14.524	3.860	5.8469 × 10^−5^	4.233
**SSEA-4**	4.915	2.297	5.8998 × 10^−5^	4.229
**CD86**	11.358	3.506	8.8496 × 10^−5^	4.053
**CD1c**	6.358	2.669	0.00039201	3.407
**CD45**	3.485	1.801	0.0012572	2.901
**CD19**	9.221	3.205	0.0022163	2.654

## Data Availability

Due to patient-sensitive data and study protocol, as well as requirements of the Institutional Review Board of the University Medical Center, the data presented in this study are available on request from the corresponding author.

## Supplementary Materials

The following supporting information can be downloaded at: https://www.mdpi.com/article/10.3390/biom16070942/s1, Figures S1–S3: Supplementary Figure S1. Uncropped Western blot images corresponding to Figure 1a. Supplementary Figure S2. Additional uncropped Western blot images for assessment of potential co-isolated contaminants. Supplementary Figure S3. Exploratory ROC curve analysis of selected sEV surface markers for discrimination of PDAC patients and healthy controls.

## Abbreviations

The following abbreviations are used in this manuscript:

CA-19.9	Cancer antigen 19.9
CD	Cluster of differentiation
EV	Extracellular vesicle
FDR	False Discovery Rate
NLC	Neuroendocrine lung cancer
NTA	Nanoparticle tracking analysis
OOB	Out of the Back
PC	Principal component
PCA	Principal component analysis
PDAC	Pancreatic ductal adenocarcinoma
sEV	Small extracellular vesicle
SOM	Self-organizing map
