# Supplementary material for: High-Dimensional Immunophenotyping of Plasma-Derived Small Extracellular Vesicles in Pancreatic Cancer: An Exploratory Proof-of-Principle Study
Source: Biomolecules. 2026 Jun 24;16(7):942. doi: 10.3390/biom16070942 (PMC13406715; doi:10.3390/biom16070942)
Supplement: Supplementary file 1 [file biomolecules-16-00942-s001.zip › biomolecules-4320219-supplementary.pdf]

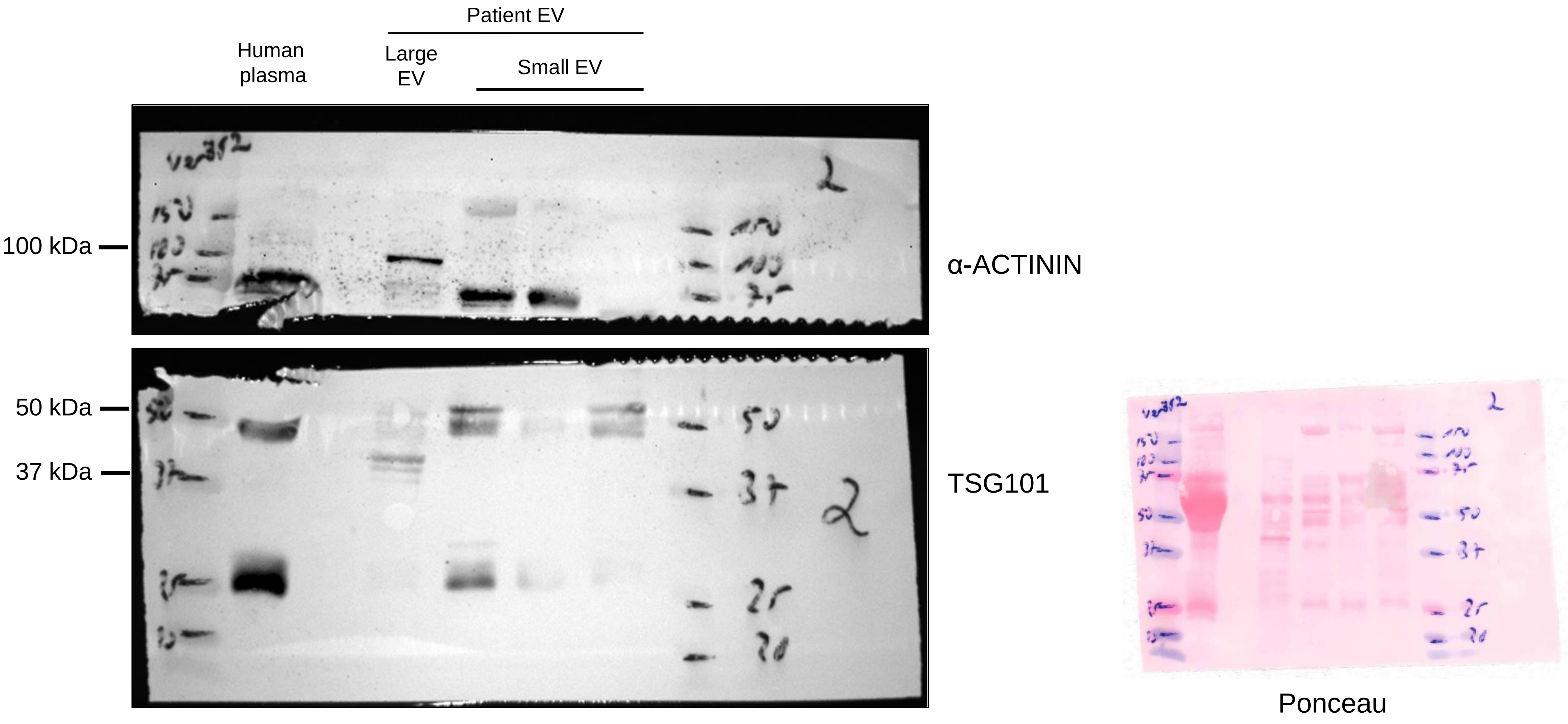

Supplementary Figure S1. Uncropped Western blot images corresponding to Figure 1a. Raw immunoblot images showing α-actinin and TSG101 staining in human plasma, large EV, and patient-derived small EV preparations. Ponceau S staining is shown as loading and transfer control. The cropped panels displayed in Figure 1a were derived from these original blot images.

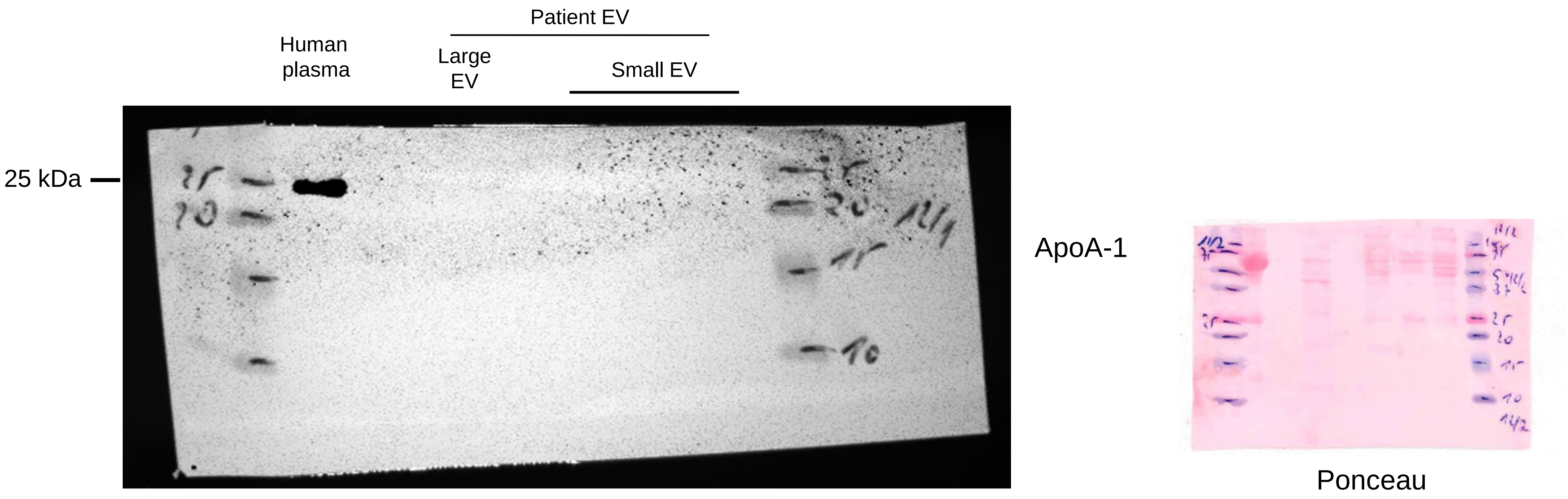

Supplementary Figure S2. Additional uncropped Western blot images for assessment of potential co-isolated contaminants. Raw immunoblot images showing ApoA-1 staining in human plasma, large EV, and patient-derived small EV preparations using the same lane and sample arrangement as in Figure 1a/Supplementary Figure S1. ApoA-1 was used as a marker of lipoprotein-associated contamination, and RacGAP1 as a marker related to cellular contamination. Ponceau S staining is shown as loading and transfer control. The corresponding cropped panels are provided in the revised manuscript as additional biochemical characterization of the sEV-enriched preparations.

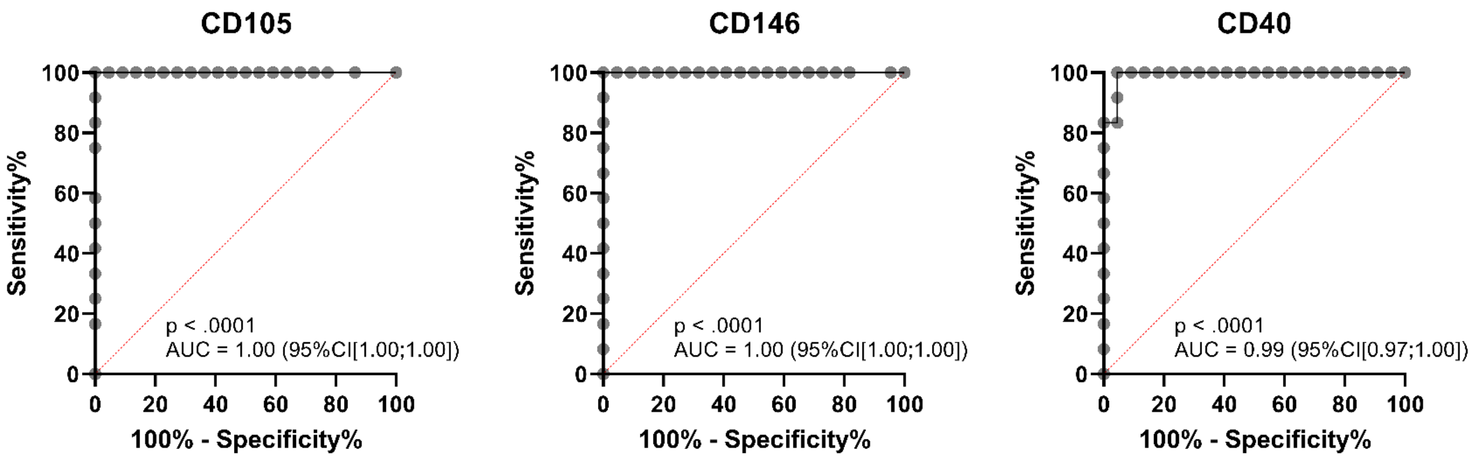

Supplementary Figure S3. Exploratory ROC curve analysis of selected sEV surface markers for discrimination of PDAC patients and healthy controls. Receiver operating characteristic (ROC) curves are shown for the three prominently altered candidate markers CD105, CD146, and CD40 based on normalized MACSPlex fluorescence intensities. The red diagonal line indicates the line of no discrimination. ROC analyses were performed descriptively to estimate the exploratory discriminatory potential of individual markers within the present cohort. Given the modest sample size and lack of an independent validation cohort, AUC values and derived performance estimates should be interpreted as hypothesis-generating and not as validated diagnostic accuracy.
